# The E3 Ubiquitin Ligase TMEM129 Is a Tri-Spanning Transmembrane Protein

**DOI:** 10.3390/v8110309

**Published:** 2016-11-15

**Authors:** Michael L. van de Weijer, Guus H. van Muijlwijk, Linda J. Visser, Ana I. Costa, Emmanuel J. H. J. Wiertz, Robert Jan Lebbink

**Affiliations:** Medical Microbiology, University Medical Center Utrecht, 3584CX Utrecht, The Netherlands; M.L.vandeWeijer@umcutrecht.nl (M.L.v.d.W.); g.h.vanmuijlwijk@uu.nl (G.H.v.M.); L.J.Visser@uu.nl (L.J.V.); A.I.CorreiadeAlmeidaCosta@umcutrecht.nl (A.I.C.)

**Keywords:** ER-associated protein degradation, ERAD, TMEM129, E3 ligase, topology, transmembrane, RING domain

## Abstract

Misfolded proteins from the endoplasmic reticulum (ER) are transported back into the cytosol for degradation via the ubiquitin-proteasome system. The human cytomegalovirus protein US11 hijacks this ER-associated protein degradation (ERAD) pathway to downregulate human leukocyte antigen (HLA) class I molecules in virus-infected cells, thereby evading elimination by cytotoxic T-lymphocytes. Recently, we identified the E3 ubiquitin ligase transmembrane protein 129 (TMEM129) as a key player in this process, where interference with TMEM129 activity in human cells completely abrogates US11-mediated class I degradation. Here, we set out to further characterize TMEM129. We show that TMEM129 is a non-glycosylated protein containing a non-cleaved signal anchor sequence. By glycosylation scanning mutagenesis, we show that TMEM129 is a tri-spanning ER-membrane protein that adopts an N_exo_–C_cyto_ orientation. This insertion in the ER membrane positions the C-terminal really interesting new gene (RING) domain of TMEM129 in the cytosol, making it available to catalyze ubiquitination reactions that are required for cytosolic degradation of secretory proteins.

## 1. Introduction

The endoplasmic reticulum (ER) is the nexus for translation of proteins destined for the secretory pathway. It is estimated that around 30% of all proteins produced in the human cell are directed towards the secretory route. The oxidative environment and folding machinery present in the ER are responsible for the proper folding of these proteins [1]. Proteins that become terminally misfolded need to be removed, as these can form larger aggregates and as such may compromise cell homeostasis and survival [2]. ER quality control mechanisms recognize misfolded proteins and redirect these towards the ubiquitin–proteasome system for degradation [3]. To this end, ER-resident proteins destined for degradation have to cross the ER membrane to reach the cytosol for proteolysis by the proteasome. This retrograde movement of proteins, called retrotranslocation or dislocation, is a key step in ER-associated protein degradation (ERAD) [4,5]. At the center of the ERAD process are multiprotein complexes that combine the various essential functions in a defined manner, namely recognition, dislocation, ubiquitination, and degradation of substrates [6].

The multiprotein complexes that facilitate ERAD generally contain multipass transmembrane E3 ubiquitin ligases harboring a really interesting new gene (RING) domain [7,8,9,10]. The RING domain of these E3 ubiquitin ligases is situated in the cytosol and serves as a docking site for an E2 ubiquitin-conjugating enzyme, which in turn catalyzes polyubiquitination of the target substrates [11]. In yeast, the main ER-resident transmembrane E3 ubiquitin ligases involved in ERAD are Hrd1p [12] and Doa10 [13]. In addition, multiprotein ERAD complexes were recently discovered to be present in the inner nuclear membrane of yeast, centered around the E3 enzymes Asi1 and Asi3 [14,15]. In contrast to yeast ERAD, mammalian ERAD shows an expansion of E3 ubiquitin ligases. Currently, ten mammalian transmembrane E3 enzymes involved in ERAD have been identified [16]: mammalian homologue of yeast Hrd1p (HRD1) [17], gp78/autocrine motility factor receptor (AMFR) [18], mammalian homologue of yeast Doa10 (TEB4/MARCHVI) [19,20], transmembrane protein 129 (TMEM129) [21,22], translocation in renal carcinoma on chromosome 8 protein (TRC8) [23,24], ring finger protein 5 (RNF5)/RMA-1 [25,26], ring finger protein 103 (RNF103)/Kf-1 [27], ring finger protein 170 (RNF170) [28], ret finger protein 2 (RFP2)/tripartite motif containing 13 (TRIM13) [29,30], and Nixin [31]. Whereas several E3 ligases, such as HRD1 and gp78/AMFR, can process many different protein substrates, other mammalian ERAD E3 enzymes have a narrower substrate range.

Several viruses exploit the ERAD pathway for the purpose of immune evasion. For example, human cytomegalovirus (HCMV) encodes the proteins US2 and US11 that exploit ERAD to induce proteasomal degradation of the antigen-presenting human leukocyte antigen (HLA) class I molecules in order to evade cytotoxic T cell responses [32,33,34]. US2 and US11 use different mechanisms to target HLA class I molecules for degradation. Whereas US2 uses TRC8 to catalyze ubiquitination [23], TMEM129 has recently been identified as the ER-resident E3 ubiquitin ligase essential for HLA class I degradation by US11 [21,22]. Prediction of membrane topology of TMEM129 suggests an N_exo_–C_cyto_ orientation with three transmembrane domains. However, predictions of membrane protein topology are not always accurate. Experimental validation of the membrane topology of ERAD E3 enzymes is key to understanding their function. Here, we experimentally map the membrane topology of the ER-resident TMEM129 using in vitro translation, truncation scanning, and glycosylation scanning mutagenesis. We demonstrate that TMEM129 is not glycosylated, does not contain disulphide bonds, and contains a non-cleaved signal-anchor sequence. In addition, we show that TMEM129 contains three transmembrane domains with an overall N_exo_–C_cyto_ orientation, thereby positioning the C-terminal RING domain in the cytosol.

## 2. Materials and Methods

### 2.1. Cell Culture and Lentiviral Infection

U937 human monocytic cells and 293T human embryonic kidney cells were obtained from American Type Culture Collection (ATCC, Manassas, VA, USA) and grown in Roswell Park Memorial Institute (RPMI) medium (Lonza, Breda, The Netherlands) supplemented with glutamine (Thermo Fischer Scientific Gibco, Waltham, MA, USA), penicillin/streptomycin (Thermo Fischer Scientific Gibco), and 10% fetal calf serum (FCS) (GE Healthcare PAA Laboratories, Eindhoven, The Netherlands). For individual gene infections using lentiviruses, the virus was produced in 293T human embryonic kidney cells seeded in 24-well plates using standard lentiviral production protocols and third-generation packaging vectors. The viral supernatant was harvested after three days post-transfection and stored at −80 °C. If required, the virus was concentrated using the Lenti-X Concentrator kit (Takara Bio Europe Clontech, Saint-Germain-en-Laye, France). For lentiviral gene infections, 50 µL of viral supernatant supplemented with 8 µg/mL polybrene (Santa Cruz Biotechnology, Heidelberg, Germany) was used to transduce approximately 20,000 U937 cells by spin infection at 1000 *g* for 2 h at 33 °C. Complete medium was added after centrifugation to reduce polybrene concentration.

### 2.2. Antibodies

Primary antibodies used in our studies were: mouse anti-CD74/Ii PIN.1 monoclonal antibody (mAb) (1/500; no. ab22603; Abcam, Cambridge, UK); mouse anti-Calnexin AF8 mAb (1/10,000; kindly provided by Michael Brenner, Harvard Medical School, Boston, MA, USA); mouse anti-transferrin receptor (TfR) H68.4 mAb (1/2500; no. 13-68xx; Thermo Fischer Scientific Invitrogen, Waltham, MA, USA); mouse anti-FLAG-M2 mAb (1/5000; no. F1804; Sigma-Aldrich, Zwijndrecht, The Netherlands); rat anti-HA 3F10 mAb (1/1000; no. 11867423001; Sigma-Aldrich Roche, Zwijndrecht, The Netherlands).

Secondary antibodies used in our studies were: F(ab′)2 goat anti-mouse IgG(H+L)-PE (1/1000; no. R0480; Agilent Dako, Middelburg, The Netherlands), goat anti-rat IgG(H+L)-PE (1/200; no. 112-116-143; Jackson Immunoresearch, Suffolk, UK), goat anti-mouse IgG(H+L)-HRP (1/10,000; no. 170-6516; Bio-Rad, Veenendaal, The Netherlands); goat anti-mouse IgG(L)-HRP (1/10,000; no. 115-035-174; Jackson Immunoresearch).

### 2.3. Plasmids

The N-terminally enhanced green fluorescent protein (eGFP)- and Myc-tagged human HLA-A2 present in the lentiviral pHRSincPPT-SGW vector was kindly provided by Dr. Paul Lehner and Dr. Louise Boyle (University of Cambridge, Cambridge, UK). For rescue and overexpression experiments, we cloned FLAG-tagged TMEM129 in a dual promoter lentiviral vector as previously described [21]. In general, the vectors carrying the cDNA used in this study also expressed ZeoR-T2A-mAmetrine under control of the human phosphoglycerate kinase (hPGK) promoter. Generation of TMEM129-FLAG and TMEM129ΔRING-FLAG vectors have been described previously [21]. Introduction of the glycosylation motifs and generation of truncation mutants was achieved by polymerase chain reaction (PCR) amplification of one or two TMEM129 fragments carrying the desired alterations and assembling these into the lentiviral ZeoR-T2A-mAmetrine vector by means of Gibson assembly (see Table 1 and Table 2 for sequence information). All constructs were verified by Sanger sequencing.

### 2.4. Flow Cytometry

For surface stainings, cells were washed in fluorescence-activated cell sorting (FACS) buffer (phosphate buffered saline (PBS), 0.5% bovine serum albumin (BSA), 0.02% sodium azide), fixed in 0.5% paraformaldehyde (PFA), and subsequently washed in FACS buffer. All subsequent staining protocols and washings were performed in FACS buffer. Afterwards, cells were subjected to flow cytometry acquisition on a FACSCanto II flow cytometer (BD Bioscience, Breda, The Netherlands). Flow cytometry data were analyzed using FlowJo software (FlowJo LLC, Ashland, OR, USA).

For intracellular stainings, two different permeabilization methods were used: Streptolysin-O (SLO) for plasma membrane permeabilization (semi-permeabilization), and saponin for full permeabilization.

Streptolysin-O permeabilization: SLO was activated by adding 4 mM dithiothreitol (DTT; Roche) and 1 mM CaCl_2_ followed by incubation at 37 °C for 10 min. Cells were kept on ice and washed once in ice-cold PBS. After resuspension in PBS, cells were incubated with SLO for 10 min. Cells were washed twice with PBS, after which cells were kept at room temperature (RT) for 20 min. Permeabilization was analyzed using Trypan Blue staining. After sufficient permeabilization, cells were fixed in 0.25% PFA and stained according to the surface staining protocol, except the washing buffer consisted of PBS supplemented with 2% FCS.

Saponin permeabilization: Cells were washed once in PBS prior to fixing in 3.7% PFA for 15 min at RT. After fixation, cells were washed once with PBS and permeabilized at RT for 10 min by adding permeabilization buffer (PBS, 2% FCS, 0.5% saponin (Sigma-Aldrich)). Subsequently, cells were stained according to the surface staining protocol, except all subsequent stainings and washings were performed in permeabilization buffer. After antibody staining, cells were washed and resuspended in PBS + 2% FCS before analysis by flow cytometry.

### 2.5. Immunoblotting

Cells were lysed in 1% Triton X-100 lysis buffer (1.0% Triton X-100, 20 mM 2-(*N*-morpholino)ethanesulfonic acid (MES), 100 mM NaCl, 30 mM Tris; pH 7.5) containing 1  mM Pefabloc SC (Roche) and 10 μM Leupeptin (Roche). Nuclei and cell debris were pelleted at 12,000 *g* for 20 min at 4 °C. Post-nuclear samples were denatured by adding Laemmli sample buffer and incubated at RT for 30 min. Proteins were separated by sodium dodecyl sulfate polyacrylamide gel electrophoresis (SDS-PAGE) and transferred to Immobilon-P polyvinylidene difluoride (PVDF) membranes (Merck Millipore, Amsterdam, The Netherlands). Membranes were probed with indicated antibodies. Reactive bands were detected by enhanced chemiluminescence (ECL; Thermo Fischer Scientific Pierce, Rockford, IL, USA), and exposed to Amersham Hyperfilm ECL films (GE Healthcare, Eindhoven, The Netherlands).

### 2.6. Immunoprecipitation

Cells were lysed in 1% Triton X-100 lysis buffer containing 1 mM Pefabloc SC (Roche) and 10 μM Leupeptin (Roche). Cell fragments were pelleted at 12,000 *g* for 20 min at 4 °C. FLAG-M2-coupled beads (Sigma-Aldrich) were pre-washed four times with lysis buffer. Next, the post-nuclear lysate was added to the beads and incubated overnight at 4 °C. Samples were then washed four times with immunoprecipitation (IP) washing buffer (1.0% Triton X-100, 100 mM NaCl, 30 mM Tris; pH 7.5). Proteins were eluted from the beads upon incubation with elution buffer (500 μg/mL FLAG-peptide (Sigma-Aldrich) in 1x Tris-buffered saline (TBS)) for 30 min on ice. Eluate was separated from the beads using a 0.45 μm Spin-X filter column (Corning Costar, Amsterdam, The Netherlands). One fraction of the eluate was used for Endo Hf/PNGase F digestion; the other was denatured using Laemmli sample buffer containing DTT for direct SDS-PAGE analysis.

### 2.7. Deglycosylation Studies

Endo H_f_: Post-nuclear lysate or eluate was denatured by adding Laemmli sample buffer and incubated at RT for 30 min, after which Endo H_f_ (New England Biolabs, Bioké, Leiden, The Netherlands) was added and incubated at 37 °C for 1 h prior to immunoblotting.

PNGase F: Eluted immunoprecipitated proteins were deglycosylated by adding G7 Reaction Buffer (New England Biolabs) and Remove-iT PNGase F (New England Biolabs), followed by incubation at 37 °C for 2 h. Note: the molecular weight of PNGase F (approximately 36 kDa) is approximately the same as that of TMEM129. To prevent distortion of the apparent molecular size of TMEM129 and putative glycosylated forms to occur, we used a removable PNGase F. Chitin Magnetic Beads (New England Biolabs) were pre-washed two times in TBS, added to the deglycosylated sample and incubated for 10 min at RT. PNGase F was removed by pelleting the beads and recovering the supernatant. Isolated proteins were denatured by adding Laemmli sample buffer, and then subjected to SDS-PAGE.

## 3. Results

### 3.1. TMEM129 Is a Non-Glycosylated Protein Lacking a Cleavable Signal Sequence and Disulphide Bonds

**Figure 1:**
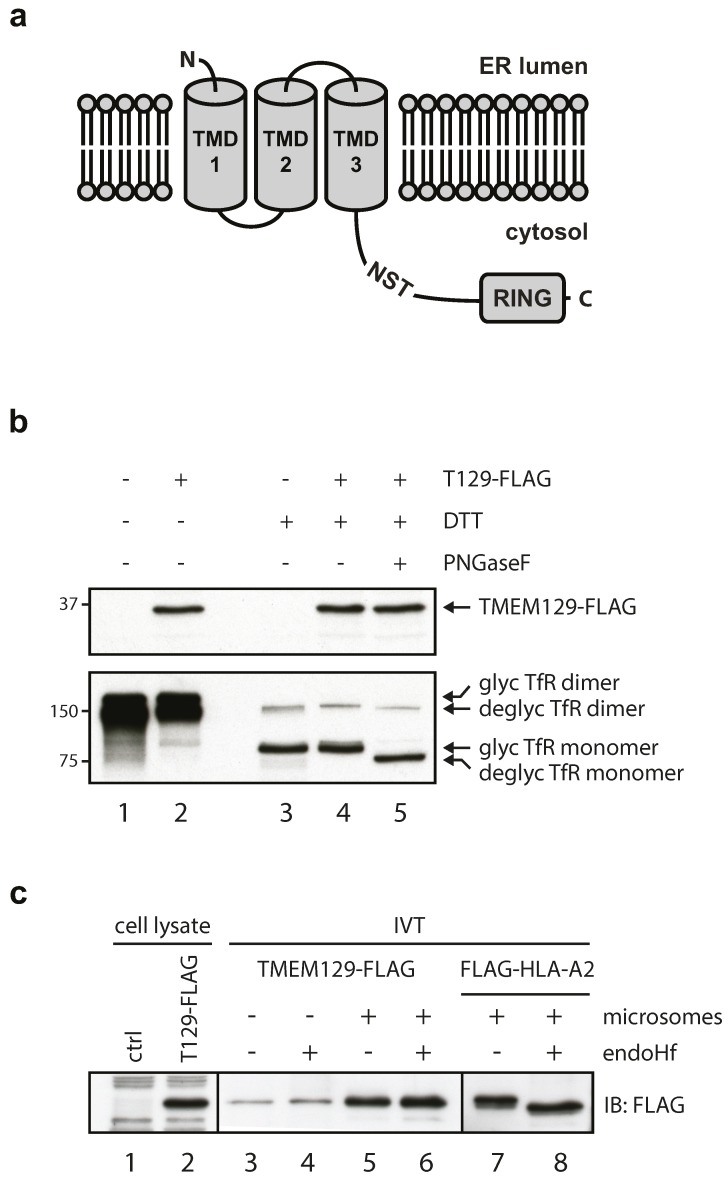
Transmembrane protein 129 (TMEM129) does not contain disulphide bonds, *N*-linked glycans, nor a cleavable signal sequence. (**a**) Predicted TMEM129 topology according to the TOPCONS membrane protein topology prediction program. Indicated are the predicted transmembrane domains (TMD) 1–3 and a putative endogenous glycosylation site (NST). ER, endoplasmic reticulum; (**b**) TMEM129 does not contain intermolecular disulphide bonds and is not glycosylated. Lysate of U937 cells expressing TMEM129-FLAG were subjected to non-reducing and reducing conditions. Reduced lysates were subjected to PNGase F digestion. Transferrin receptor (TfR) served as a control for both reducing and deglycosylating conditions. TMEM129 and TfR were visualized by immunoblotting with specific antibodies. Glycosylated and deglycosylated proteins are indicated; (**c**) In vitro translation of TMEM129-FLAG in the absence and presence of microsomes shows that TMEM129 does not contain a cleavable signal peptide. Simultaneous deglycosylation revealed that TMEM129 is not glycosylated. As a positive control, HLA-A2 was translated in vitro in the presence of microsomes and subjected to endo Hf treatment. TMEM129 and HLA-A2 were visualized by immunoblotting with a FLAG-reactive antibody. Experiments were performed three times, of which one representative experiment is shown.

TMEM129 is a transmembrane protein of 362 amino acid residues, localized to the ER [21,22], and has been predicted to contain three transmembrane domains according to the TOPCONS membrane protein topology prediction server (Figure 1a) [35]. One putative glycosylation site consisting of the amino acid residues NST is present at amino acid position 229–231. According to membrane topology predictions, this putative glycosylation site is expected to reside in the cytosol. Hence, the glycosylation machinery should not have access to this site, and glycosylation will probably not occur. To verify this, lysates were prepared from U937 cells expressing TMEM129-FLAG and subjected to deglycosylation using PNGase F (Figure 1b). No shift in molecular size was observed for TMEM129, indicating that TMEM129 is a non-glycosylated protein (Figure 1b, compare lanes 4 and 5). Transferrin receptor was successfully deglycosylated. Also, the mobility of TMEM129 was similar in non-reducing and reducing conditions, indicating that TMEM129 does not contain intermolecular disulphide bonds (Figure 1b, lanes 2 and 4).

The first transmembrane domain is predicted to span amino acid residues 7 through 28, but whether these residues represent a cleavable signal sequence (aa 1–28) or a non-cleavable signal anchor (aa 7–28) remains unknown. To study this, TMEM129-FLAG was translated in vitro in the absence (Figure 1c, lane 3 and 4) or presence (lane 5 and 6) of microsomes. As we did not observe a difference in the apparent TMEM129-FLAG molecular size between both conditions, the E3 ligase appears to contain a non-cleavable signal anchor (Figure 1c, lanes 3 and 5). Also, endo Hf-mediated digestion of the samples did not alter the molecular size of TMEM129, again showing that TMEM129 is not glycosylated and hence likely correctly inserted into the microsomes (Figure 1c, lanes 5 and 6). As a positive control for insertion into the microsomes, HLA-A2 was translated in vitro in the presence of microsomes (Figure 1c, compare lanes 7 and 8). Upon endo Hf-mediated digestion (lane 8), a lower molecular size species became apparent, showing that HLA-A2 was efficiently and correctly inserted into the microsomes. These findings indicate that TMEM129 lacks *N*-linked glycans and intermolecular disulphide bonds, and is inserted into the membrane through a non-cleaved signal anchor.

### 3.2. TMEM129 Is Localized in the *Endoplasmic Reticulum* Membrane in an N_exo_–C_cyto_ Orientation

**Figure 2:**
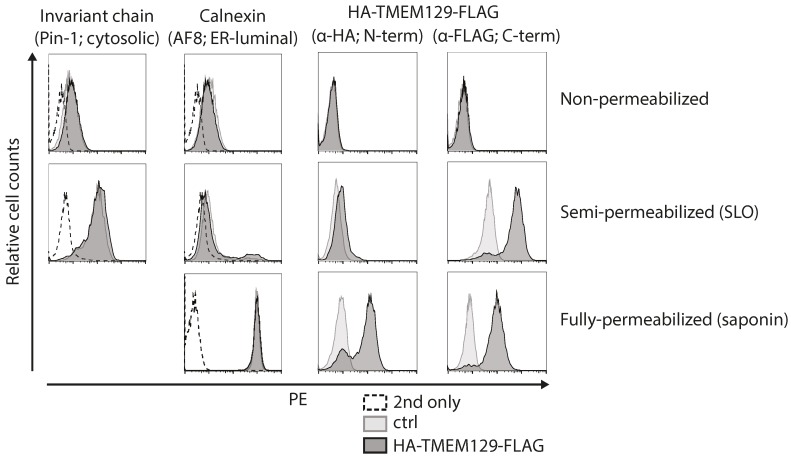
The N-terminus of TMEM129 is localized in the ER lumen, the C-terminus in the cytosol. U937 cells transduced with either HA-TMEM129-FLAG or vector control (ctrl) were either left untreated (non-permeabilized), semi-permeabilized using streptolysin-O (SLO), or fully-permeabilized using saponin. The termini of HA-TMEM129-FLAG were detected using HA- or FLAG-reactive antibodies respectively, after which flow cytometric analysis was performed. As a control for semi-permeabilization, cells were stained with a monoclonal antibody directed against a cytosolic epitope of the invariant chain (Ii) or were incubated with the secondary antibody only (2nd only). As a control for full-permeabilization, cells were stained with a monoclonal antibody directed against an ER-luminal epitope of calnexin or they were incubated with the secondary antibody only. Experiments were performed three times, of which one representative experiment is shown.

Next, we set out to experimentally map the orientation of TMEM129 by determining the subcellular localization of its N- and C-termini. As the N-terminus of TMEM129 contains a non-cleaved signal-anchor sequence, an epitope tag could be inserted at both the N- and C-terminus (HA- and FLAG-tag, respectively) to allow assessment of the orientation of TMEM129 within the ER membrane. Upon stable introduction of HA-TMEM129-FLAG in U937 cells, cells were subjected to selective permeabilization using the pore-forming toxin SLO, or the detergent saponin. Through carefully timed application, activation, and washing, SLO was used to selectively permeabilize the plasma membrane, leaving the ER membrane intact. The detergent saponin was used to permeabilize both the plasma membrane and the ER membrane. By using both permeabilization methods prior to staining with HA- and FLAG-specific antibodies, the exact subcellular localization of the N- and C-terminus can be assessed. As a positive control for proper membrane permeabilization, monoclonal antibodies were used that recognize either a cytosolic epitope of the invariant chain (Ii) or an ER-luminal epitope of the ER-resident calnexin. Indeed, cells were properly permeabilized, as anti-calnexin antibodies only stained saponin-treated cells, but not SLO-treated cells, whereas anti-Ii antibodies did react with SLO-treated cells (Figure 2, left panels). Flow cytometric analysis of HA-TMEM129-FLAG revealed that anti-HA antibodies recognized saponin-treated, but not SLO-treated, cells (Figure 2). This indicates that the N-terminus is located in the ER lumen (Figure 2). On the other hand, the FLAG-reactive antibody reacted in both SLO- and saponin-permeabilized cells, suggesting that the C-terminus is present in the cytosol. These data show that TMEM129 adopts an overall N_exo_–C_cyto_ orientation.

### 3.3. The Cytosolic Tail of TMEM129 Is Essential for Activity

**Figure 3:**
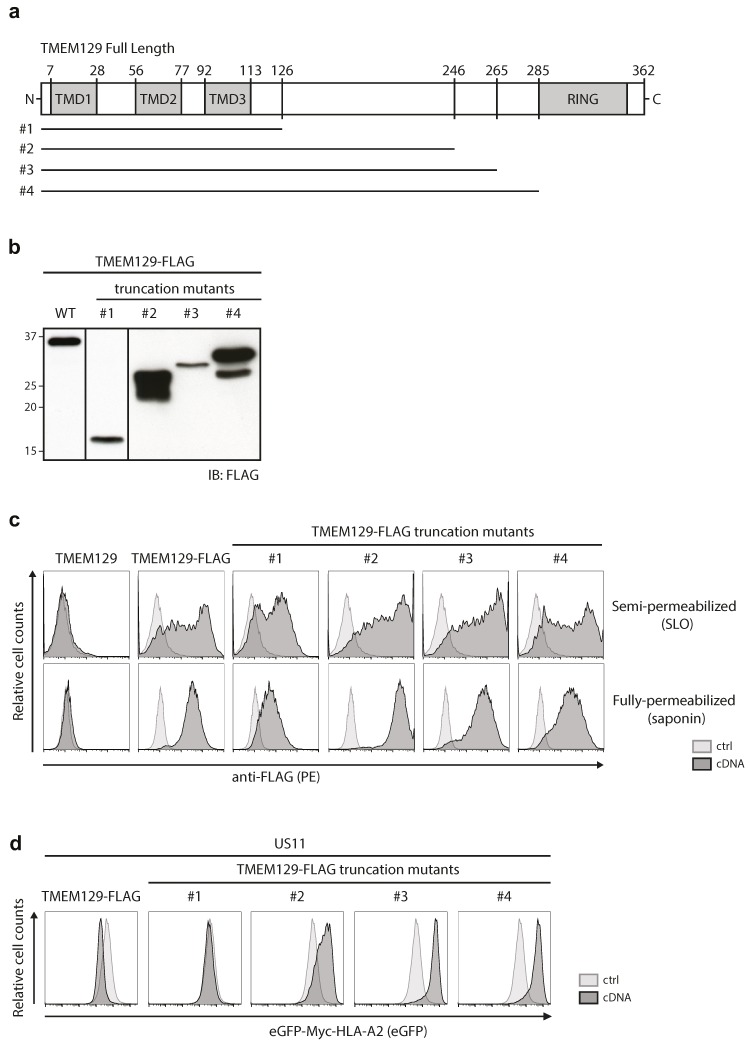
The cytosolic tail of TMEM129 is essential for activity. (**a**) Schematic representation of TMEM129 truncation mutants; (**b**) Truncation mutants of TMEM129 were expressed in U937 cells, after which the lysate was prepared and subjected to immunoblotting using a FLAG-reactive antibody. The full-length TMEM129-FLAG protein is indicated as wild-type (WT); (**c**) TMEM129 truncation mutants are properly inserted in the ER membrane. U937 cells expressing TMEM129-FLAG truncation mutants were semi-permeabilized using SLO, or fully-permeabilized using saponin. The C-terminus of the truncation mutants was detected using a FLAG-reactive antibody, after which flow cytometric analysis was performed; (**d**) Deletion of the entire TMEM129 cytosolic tail interfered with US11-mediated HLA class I downregulation whereas deletion of the RING domain resulted in a dominant-negative phenotype. Indicated truncation mutants of TMEM129-FLAG were expressed in U937 cells, co-expressing eGFP-Myc-HLA-A2 and HCMV US11. Flow cytometric analysis was performed to assess the total levels of eGFP-Myc-HLA-A2. Experiments were performed three times, of which one representative experiment is shown.

We next generated TMEM129-FLAG truncation mutants to assess which regions of the protein are important for its activity. We generated multiple truncation variants, in which increasing parts of the predicted cytosolic domain of TMEM129 were deleted. Although several deletion variants were not expressed (data not shown), four mutants (Figure 3a) were readily detected upon stable transduction in U937 cells (Figure 3b). To assess whether these proteins were properly inserted in the ER membrane, the localization of their C-termini were assessed by selective permeabilization followed by anti-FLAG antibody stains. In both SLO- and saponin-permeabilized cells, the C-terminal FLAG-tagged truncation mutants #1–4 were detected, indicating that their C-termini were indeed still present in the cytosol (Figure 3c).

We previously showed that expression of full-length TMEM129 enhanced HLA class I downregulation in eGFP-Myc-HLA-A2 and US11 expressing cells ([21] and Figure 3d, left panel), whereas expression of a RING-less TMEM129 mutant (truncation mutant #4) resulted in a dominant-negative phenotype, efficiently rescuing eGFP-Myc-HLA-A2 expression ([21] and Figure 3d right panel). Correspondingly, deletion of the RING domain in truncation mutants #2 and #3 resulted in a dominant-negative phenotype. Truncation mutant #1, however, completely lost E3 ligase activity and also did not act as a dominant-negative variant. Hence, the region between amino acids 126–246 is responsible for the switch to the dominant-negative phenotype, and it is therefore suggestive that this area is involved in the recruitment of interaction partners to aid in the US11-mediated HLA class I dislocation event. 

### 3.4. TMEM129 Contains Three Transmembrane Domains

**Figure 4:**
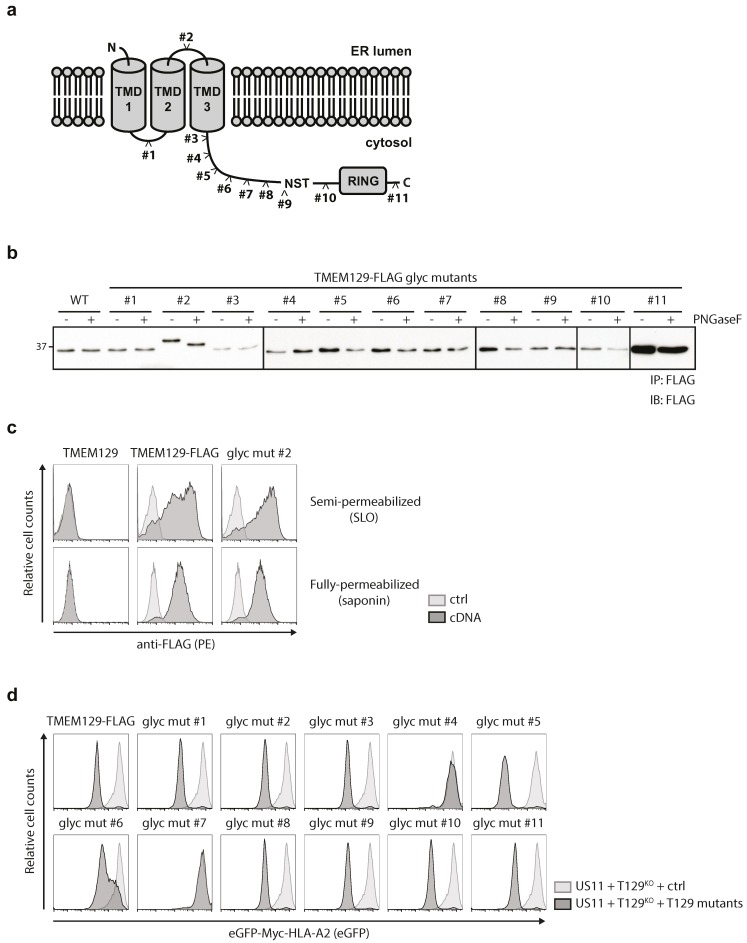
TMEM129 is a tri-spanning ER membrane protein. (**a**) Glycosylation motif insertion mutants of TMEM129 (#1–11) were generated as indicated; (**b**) The loop between transmembrane domain (TMD) 2 and TMD3 is located inside the ER lumen. TMEM129-FLAG glycosylation mutants were expressed in U937 cells, and subjected to immunoprecipitation using anti-FLAG-coupled beads. Eluted proteins were then subjected to PNGase F digestion. After PNGase F was removed, TMEM129-FLAG mutants were detected using a FLAG-reactive antibody. This experiment was performed two times, of which one representative experiment is shown; (**c**) The C-terminal orientation of glycosylation mutant #2 is unaltered. U937 cells expressing TMEM129-FLAG or glycosylation mutant #2 were semi-permeabilized using SLO, or fully-permeabilized using saponin. The respective C-termini were detected using a FLAG-reactive antibody, after which the flow cytometric analysis was performed; (**d**) Most, but not all, glycosylation insertions did not affect TMEM129 activity. TMEM129-FLAG glycosylation mutants were expressed in TMEM129-null U937 cells, co-expressing eGFP-Myc-HLA-A2 and HCMV US11. Flow cytometric analysis was performed to assess the total levels of eGFP-Myc-HLA-A2. Experiments were performed three times, of which one representative experiment is shown.

To study the topology of TMEM129 in more detail, *N*-linked glycosylation acceptor sequences were inserted at various sites throughout the protein. As *N*-glycosylation only occurs in the ER lumen, evaluation of the glycosylation status of these mutants allows for an accurate assessment of the subcellular localization of these motifs which aids in determining the number of transmembrane domains for TMEM129. We constructed 11 different TMEM129 glycosylation mutants, in which the NATE glycosylation consensus motif was introduced (Figure 4a). In addition, these TMEM129 glycosylation mutants were C-terminally fused to a FLAG-tag to facilitate detection by flow cytometry and immunoblotting experiments. The mutants, stably expressed in U937 cells, were immunoprecipitated using anti-FLAG coupled beads. Next, immunoprecipitates were left untreated or were incubated with PNGase F to cleave all *N*-linked glycans. Upon Western blotting for TMEM129-FLAG, only mutant #2 was sensitive to PNGase F treatment resulting in a shift in apparent molecular weight, whereas the other mutants were non-reactive (Figure 4b). As the putative ER-luminal loop between the predicted transmembrane domain 2 and 3 was rather short, we introduced the NATE consensus sequence as part of a 16-amino acid insertion, explaining the increased size of the protein as compared to the other mutants. Importantly, the TMEM129-FLAG mutant #2 was correctly inserted into the ER membrane, as the C-terminal FLAG-sequence was present in the cytosol (Figure 4c).

We next assessed the functionality of the TMEM129 glycosylation mutants in US11-expressing cells (Figure 4d). The TMEM129 glycosylation mutants were stably expressed in U937 TMEM129-null cells co-expressing eGFP-Myc-HLA-A2 and the HCMV protein US11. Reconstitution of a functional TMEM129 protein restored US11-mediated HLA class I downregulation in TMEM129-null cells, as measured by flow cytometry (Figure 4d, top left panel). Glycosylation mutant #2 was still functional, as it could rescue the TMEM129-null phenotype, suggesting that the topology of TMEM129 mutant #2 is unaltered. Only glycosylation mutants #4 and #7 did not rescue the TMEM129-null phenotype, indicating that insertion of the glycosylation acceptor sequence either disrupts protein topology or interferes with TMEM129 activity by altering an essential domain of the E3 ligase. In conclusion, our data show that the N-terminus of TMEM129 resides in the ER, the glycosylation site of mutant #1 is present in the cytosol, the glycosylation site of mutant #2 is localized to the ER lumen, and the glycosylation sites for mutants #3 through #11 together with the C-terminus reside in the cytosol. Hence, our data support a model in which TMEM129 is inserted in the ER membrane by three transmembrane domains.

## 4. Discussion

The topology of many proteins involved in ERAD is poorly studied and current models are often based on prediction software that may be incorrect. Therefore, experimental validation of protein topology is essential for understanding their function. Indeed, the multipass transmembrane protein Derlin-1 was predicted to contain four transmembrane domains [36,37,38], although later studies showed that this important ERAD factor contains six transmembrane domains, consistent with its homology to rhomboid family members [39]. Additionally, the topology and number of transmembrane domains of gp78/AMFR is still a matter of debate as, depending on the algorithm used, the estimated number of transmembrane domains ranges from five to seven [40]. Using HCMV US11-mediated HLA class I downregulation as a model [21,22], TMEM129 has been recently identified as an ER-resident E3 ubiquitin ligase. In the present study, we have determined the general characteristics and the topology of TMEM129.

Our data show that TMEM129 does not contain *N*-linked glycans, or disulphide bonds. Moreover, TMEM129 does not contain a cleavable signal peptide, suggesting that the first transmembrane domain functions as a signal-anchor sequence. Topological analysis of the N- and C-termini using flow cytometry revealed that the N-terminus is in the ER-lumen and the C-terminus in the cytosol. Hence, the subcellular location of the C-terminus is consistent with the presence of a functional RING domain in the cytosolic C-terminus that catalyzes the ubiquitination reaction.

We have constructed a set of C-terminal truncation mutants to map the ligase activity of TMEM129. We inferred that all mutants were properly inserted into the ER membrane, as no changes were made to the TMDs and their C-terminal FLAG-tags were localized in the cytosol (Figure 3c). However, besides the expected full-length products, cells transduced with mutants #2 and #4 also expressed a smaller, less abundant, species (Figure 3b). As the TMEM129 truncation mutants were tagged at the C-terminus, and the Western blot was stained with a monoclonal antibody directed against this tag, it is possible that the lower molecular weight species were N-terminal truncation variants that may (partially) lack the first TMD. As this TMD could function as an anchor sequence, these proteins may not have been properly inserted into the ER membrane, which could have affected their activity. However, as the majority of TMEM129 in cells transduced with these two mutants migrated at the correct molecular weight (Figure 3b), and no apparent altered staining patterns were observed between these mutants and the full-length FLAG-tagged TMEM129 (Figure 3c), we assume that the impact of these smaller TMEM129 proteins are limited.

Using the TOPCONS webserver, TMEM129 was predicted to have three transmembrane domains which was confirmed in our study. The predicted transmembrane regions are located at amino acid positions 6–26 (TMD1), 56–76 (TMD2), and 95–115 (TMD3). Based on this prediction, the loop between TMD1 and TMD2 (loop 1) would span 28 amino acids and the loop between TMD2 and TMD3 (loop 2) would span 17 amino acids. In general, luminal loops containing *N*-linked oligosaccharides have a minimum size of ±25–30 residues, with the glycosylation acceptor site located at least 10 residues away from a predicted transmembrane domain [41,42] due to steric hindrance constraint of the oligosaccharyltransferase with the ER membrane and the glycosylation acceptor sequence during translation [43]. Hence, glycosylation consensus motifs present in short loops might fail to be glycosylated which can be prevented by artificially extending the length of the loop [42]. Thus, for putative glycosylation to occur in glycosylation mutant #2 (containing an inserted glycosylation acceptor sequence in loop 2), the NATE consensus sequence was flanked by eight additional amino acid residues on both sides. The resulting protein is 18 amino acids longer, which was reflected by immunoblotting data. The extension of loop 2 did not influence the functionality of the mutant, as it could still rescue US11-mediated HLA class I downregulation in TMEM129-null cells. However, two other glycosylation mutants, #4 and #7, could not rescue US11-mediated HLA class I downregulation. These mutants were not glycosylated; hence it is likely that the insertion of a glycosylation site disrupted TMEM129 function or a protein–protein interaction essential for US11-mediated HLA class I downregulation.

Although it has been established that TMEM129 is essentially involved in retrograde transport and degradation of HLA class I molecules [21,22], its exact role in this process remains to be determined. An important, yet unresolved, question is how ER-resident degradation substrates cross the ER membrane. This pertains to membrane proteins such as HLA class I molecules, but also to soluble ER proteins destined for degradation in the cytoplasm. Most likely, these proteins migrate back into the cytosol through a channel [44]. Using US2-mediated degradation of HLA class I molecules as a model, a role has been proposed for the Sec61 channel, generally involved in protein import into the ER [32]. This possibility is supported by studies using other proteins as dislocation substrates [45,46,47,48,49,50,51,52,53,54].

For US11-mediated degradation of HLA class I molecules, no indications were found for a role of the Sec61 channel in dislocation of the substrate; several other multipass membrane proteins have been proposed to fulfill this function. These include the Derlins, multipass membrane proteins widely involved in ERAD [37,38,39,55,56,57]. Derlin-1 has been shown to contain six transmembrane domains [39] and the protein is involved in the degradation of HLA class I molecules in the context of US11, but not US2 [37,38]. In addition, several E3 ligases that are involved in ERAD are multipass membrane proteins and may therefore be part of the dislocation channel. Yeast Hrd1p, for example, contains six transmembrane domains [58]. Human HRD1 likely has an identical topology [59] and is involved in degradation of misfolded HLA class I molecules in the absence of US2 and US11 [60,61]. Experiments involving the in vitro reconstitution of retrotranslocation using proteoliposomes and purified *Saccharomyces cerevisiae* proteins suggest that Hrd1p forms a ubiquitin-gated protein-conducting channel [62,63]. TEB4 and its yeast homolog Doa10 have been reported to encompass fourteen TMDs [64]. For gp78/AMFR, the number of TMDs has been predicted to be between five and seven [40]. Although TMEM129 only contains three TMDs, this E3 ligase may still form part of the dislocation channel, especially if TMEM129 forms multimers. If TMEM129 indeed occurs as a multimeric complex, our experiments indicate that this does not rely on the formation of disulfide bonds.

In conclusion, TMEM129 adopts an N_exo_–C_cyto_ orientation with three transmembrane domains and a C-terminal cytosolic RING domain. This topology is in agreement with its function as an E3 ubiquitin ligase involved in the degradation of ER-resident proteins.

## Figures and Tables

**Table 1:** Primer sequences for glycosylation mutants.

Glyc Mutant	Forward or Reverse	Sequence 5′–3′
Backbone	Forward	TGAGCTAGCAGTATTAATTAACCAC
	Reverse	ATGACTAAGCTAGTACCGGTTAG
#1 Ser43 NAT	Forward	GTCGGGCTGGCTGGGCAGCAACGCAACAGAGGACGCCGCCTTCGTG
	Reverse	CACGAAGGCGGCGTCCTCTGTTGCGTTGCTGCCCAGCCAGCCCGAC
#2 Ala85 GAAGGAANATEGAAEGAAGG	Forward	ACCGCCAGCTGCACCTCCTGCCGCGCC**CTCTGTTGCGTT**AGCTGCACCTCCTGCCGCGCCGGCGTGGAGCCGCTTTTCTG
	Reverse	GGCGCGGCAGGAGGTGCAGCT**AACGCAACAGAG**GGCGCGGCAGAAGGTGCAGCTGGCGGTCTCAGCCAGGCCCCTGAG
#3 Asp117 NATE	Forward	CTACTACTGGTCCCGTGACAACGCAACAGAGCGGTGGGCCTGCCACCC
	Reverse	GGGTGGCAGGCCCACCGCTCTGTTGCGTTGTCACGGGACCAGTAGTAG
#4 Ala125 NATE	Forward	GCCTGCCACCCACTGGCGAACGCAACAGAGCGCACCCTGGCCCTCTACG
	Reverse	CGTAGAGGGCCAGGGTGCGCTCTGTTGCGTTCGCCAGTGGGTGGCAGGC
#5 Ala140 NATE	Forward	CACAGTCTGGCTGGCAGGCTAACGCAACAGAGGTTGCCTCCTCTGTCAACAC
	Reverse	GTGTTGACAGAGGAGGCAACCTCTGTTGCGTTAGCCTGCCAGCCAGACTGTG
#6 Ile152 NAT	Forward	CACTGAGTTCCGGCGGATTAACGCAACAGAGGACAAGTTTGCCACCGGTG
	Reverse	CACCGGTGGCAAACTTGTCCTCTGTTGCGTTAATCCGCCGGAACTCAGTG
#7 Trp170 NATE	Forward	GTGATTGTGACAGACACGTGGAACGCAACAGAGGTGATGAAGGTAACCACCTAC
	Reverse	GTAGGTGGTTACCTTCATCACCTCTGTTGCGTTCCACGTGTCTGTCACAATCAC
#8 Val191 NA	Forward	GGACGTGCACCTGACTGTGAACGCAACGGAGTCTCGGCAGCATG
	Reverse	CATGCTGCCGAGACTCCGTTGCGTTCACAGTCAGGTGCACGTCC
#9 Ser230 NA	Forward	CTTTGACATCTGGCTGAACTCCAACGCAACTGAGTACGGGGAGCTCTG
	Reverse	CAGAGCTCCCCGTACTCAGTTGCGTTGGAGTTCAGCCAGATGTCAAAG
#10 Ser275 NATE	Forward	GAGGTCAACCCGGCCTACTCAAACGCAACAGAGGTGCCCAGCAGCCAGGAG
	Reverse	CTCCTGGCTGCTGGGCACCTCTGTTGCGTTTGAGTAGGCCGGGTTGACCTC
#11 Phe363 NATE	Reverse	ATGACTAAGCTAGTACCGGTTAGGATGCATTCACTTGTCGTCATCGTCTTTGTAGTCTTCCTCTGTTGCGTTGAAGCGCACGGTGCACAC

This table contains the sequences of primers used with TMEM129-FLAG as a template. The ‘Glyc Mutant’ column displays the glycosylation mutant number, the amino acid (aa) residue immediately preceding the glycosylation acceptor sequence that was inserted, and the inserted amino acids. In the ‘Sequence 5′–3′’ column, the inserted nucleotides encoding for these are underlined. In mutant #2, the sequence highlighted in bold is the inserted glycosylation consensus sequence.

**Table 2:** Primer sequences for truncation mutants.

Truncation Mutant	Forward or Reverse	Sequence 5′–3′
#1	Reverse	ATGACTAAGCTAGTACCGGTTAGGATGCATTCACTTGTCGTCATCGTCTTTGTAGTCTTCGAAGGCAAATGTCTCCAGG
#2	Reverse	ATGACTAAGCTAGTACCGGTTAGGATGCATTCACTTGTCGTCATCGTCTTTGTAGTCTTCGAACCTGCGGATGGGTGCCCG
#3	Reverse	ATGACTAAGCTAGTACCGGTTAGGATGCATTCACTTGTCGTCATCGTCTTTGTAGTCTTCGAATGGCGAGAGCTCATGCTGC
#4	Reverse	ATGACTAAGCTAGTACCGGTTAGGATGCATTCACTTGTCGTCATCGTCTTTGTAGTCTTCGAAGCGCGCCAGTGGGTGGC

This table contains the sequences of primers used for generation of truncation mutants (#1–4, ‘Truncation Mutant’ column), with TMEM129-FLAG as a template.
